# Subcutaneous switching suture technique for hernia defect closure during laparoscopic ventral and incisional hernia repair

**DOI:** 10.1111/ases.12839

**Published:** 2020-07-28

**Authors:** Daisuke Morioka, Yusuke Izumisawa, Norio Ohyama, Kazuya Yamaguchi, Nobutoshi Horii, Fumio Asano, Masaru Miura, Yoshiki Sato

**Affiliations:** ^1^ Department of Surgery Yokohama Ekisaikai Hospital Yokohama Japan

**Keywords:** hernia defect closure, laparoscopic ventral and incisional hernia repair, suturing technique

## Abstract

**Introduction:**

A vertical penetration of the thread through the abdominal wall for the hernia defect closure in laparoscopic ventral/incisional hernia repair (LVIHR) is difficult especially in the large defect cases when applying the existing techniques.

**Materials:**

Sixteen LVIHRs were performed using the suture technique for defect closure we newly developed.

**Surgical technique:**

With the subcutaneous switching, our technique only requires the suture‐passer and easily enables the vertical penetration of the thread through the abdominal muscular wall even in the large defect cases.

**Discussion:**

The defect closure in LVIHR tends to be complicated in the large defect cases. Thus, we devised this technique for the easy, reliable, and firm closure even in the large defect cases. Although the sample size was currently very small, we consider that the favorable outcomes have been obtained through our technique because any noticeable complications, such as mesh bulging or recurrence, have not been observed currently.

## 1 INTRODUCTION

Although the clinical significance of hernia defect closure remains controversial,^1^, ^2^, ^3^, ^4^, ^5^, ^6^, ^7^, ^8^, ^9^ the intraperitoneal‐onlay‐mesh repair with hernia defect closure (IPOM‐plus) in laparoscopic ventral and incisional hernia repair (LVIHR) has become the common technique in many institutions.^2^, ^3^, ^4^, ^5^, ^6^, ^7^, ^8^, ^9^ The method for defect closure in the IPOM‐plus is roughly divided into the following two methods: the percutaneous suture using a suture‐passer and the intracorporeal suture. Generally, the former is easy^3^, ^8^ but the latter is less easy.^2^, ^4^, ^5^, ^6^, ^7^, ^8^, ^9^

In the conventional method of percutaneous suture, the thread for closing the defect is entered into the abdomen using the suture‐passer through the small incision on the midline of the hernia defect. Subsequently, the thread in the abdomen is exteriorized through the same incision using the suture‐passer. In cases of large defect, therefore, the direction of the suture‐passer penetrating the abdominal wall has to be oblique, that is, not vertical.^3^, ^8^ In such occasions, the thread for defect closure is likely passed only through the posterior fascia of the rectus muscle: that is, the thread cannot penetrate the anterior fascia because of the oblique direction of the suture‐passer. As such, the approximation of the fascial edges of the defect may be likely to dehisce because of the lack of the anterior fascia in the stitches, leading to mesh bulging or hernia recurrence.^2^, ^3^, ^4^, ^5^, ^6^, ^7^, ^8^, ^9^

In the intracorporeal suture, the vertical penetration of the thread through the anterior and posterior fascias can be reliably performed.^2^, ^4^, ^5^, ^6^, ^7^, ^8^, ^9^ However, this technique requires some technical expertise, its dedicated devices, and the thread‐attached‐to‐the‐needle, which is less cost‐effective than the needleless thread.

We herein introduce an easy and reliable pure‐percutaneous technique for the IPOM‐plus, which only requires the suture‐passer and easily enables the vertical penetration of the thread through the anterior and posterior fascias even in large defect cases.

### 1.1 Surgical technique

Written informed consent that guaranteed anonymity was provided to all study participants. This study was approved by the Institutional Review Board (IRB approval No. YEH2020‐S‐01) and was Declaration of Helsinki compliant.

**FIGURE 1:**
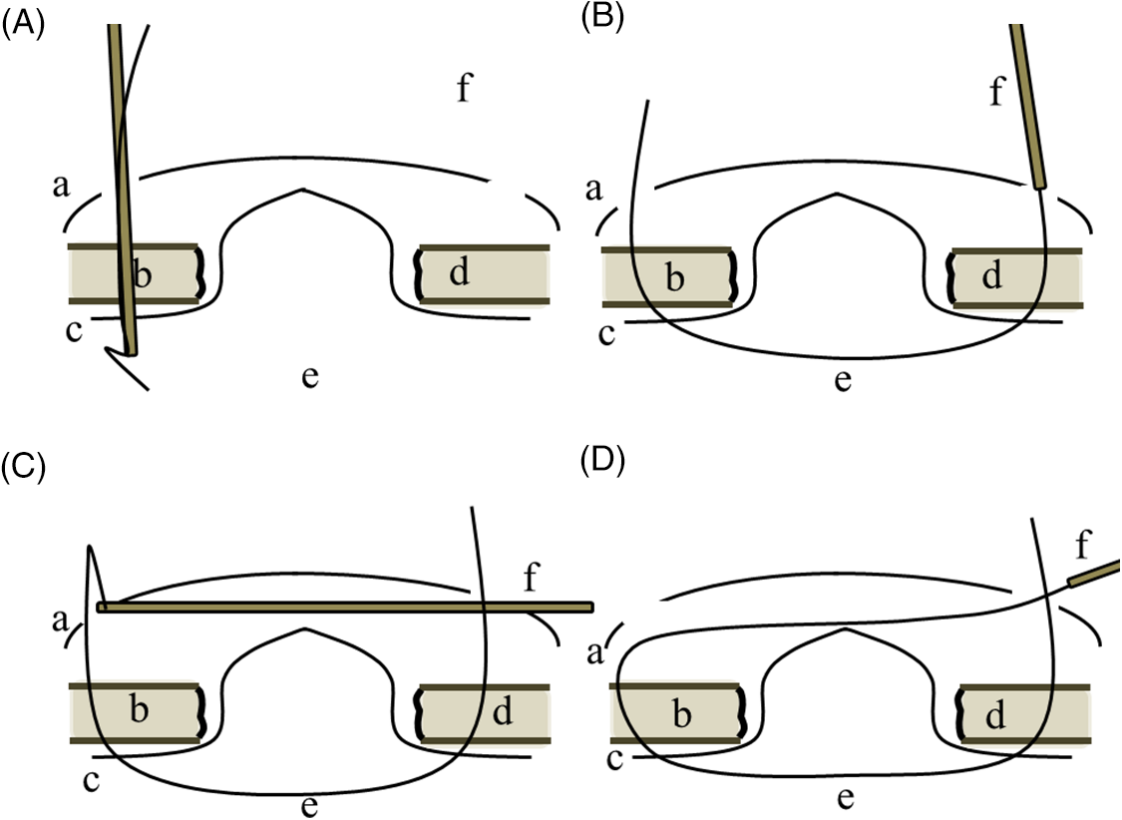
Schema of the subcutaneous switching suture technique. A, A needleless thread was passed through the incision into the abdomen using the suture‐passer, in the direction vertical to the abdominal muscular wall. B, Then, the thread in the abdomen was exteriorized through the incision on the contralateral end of the vertical line using the suture‐passer. Then, each end of the thread was exteriorized from both ends of the vertical line. C, Special attention was paid to the direction of the suture‐passer penetrating the abdominal wall. After that, the suture‐passer was passed subcutaneously from one end to the contralateral end of the vertical line. D, Then, the thread of the contralateral end was passed using the suture‐passer to one end, through which the suture‐passer was entered toward the contralateral end. Then, both ends of the thread were exteriorized through the same incision. (a, skin; b, right edge of the hernia defect; c, parietal peritoneum; d, left edge of the defect; e, thread; f, suture‐passer)

**FIGURE 2:**
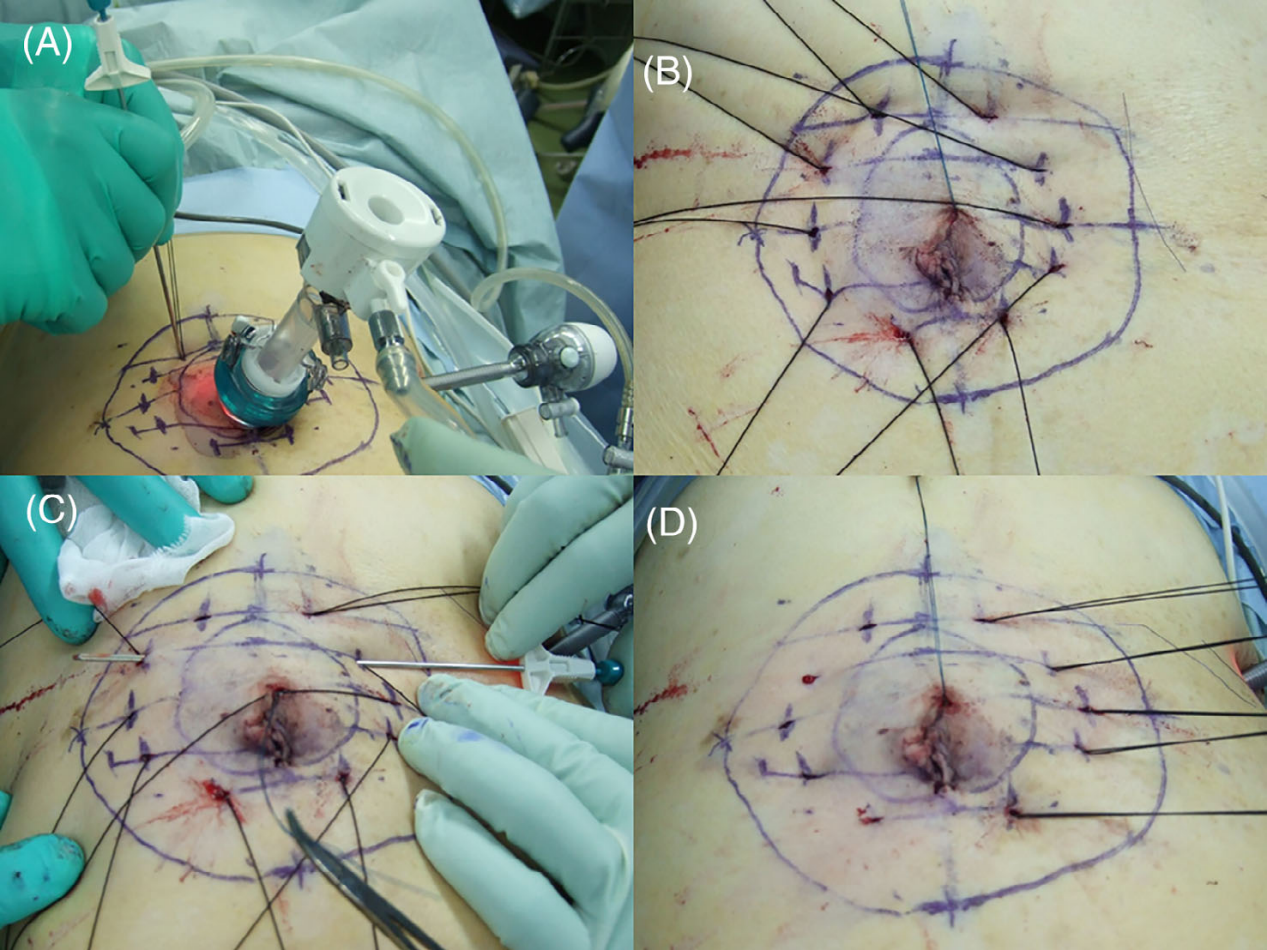
Procedures of the subcutaneous switching suture technique. A, A needleless thread was passed through the incision into the abdomen using the suture‐passer, in the direction vertical to the abdominal muscular wall. B, Then, the thread in the abdomen was exteriorized through the incision on the contralateral end of the vertical line using the suture‐passer. Then, each end of the thread was exteriorized from both ends of the vertical line. C, Special attention was paid to the direction of the suture‐passer penetrating the abdominal wall. After that, the suture‐passer was passed subcutaneously from one end to the contralateral end of the vertical line. D, Then, the thread of the contralateral end was passed using the suture‐passer to one end, through which the suture‐passer was entered toward the contralateral end. Then, both ends of the thread were exteriorized through the same incision. After switching all threads, a mesh coated with an absorbable hydrogel barrier was inserted into the abdomen through a port. Then, threads are ligatured tightly, achieving the defect closure, after which the mesh was spread and fixed to the abdominal wall

**FIGURE 3:**
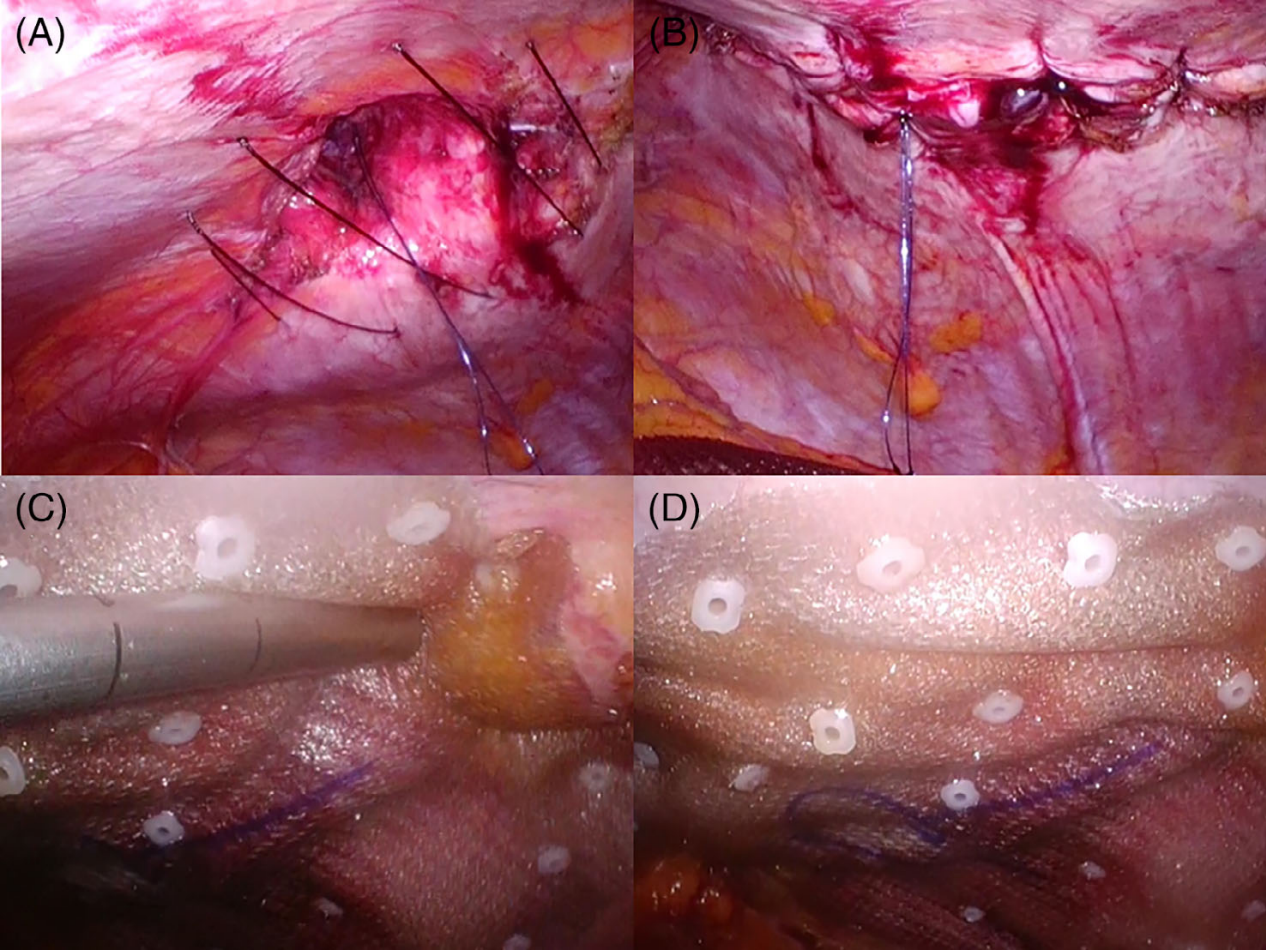
Procedures after subcutaneous switching. A, After switching all threads, a mesh coated with an absorbable hydrogel barrier was inserted into the abdomen through a port. B, Subsequently, threads are ligatured tightly, achieving the defect closure. C, D, Then, the mesh was spread and fixed to the abdominal wall

The details are shown in Figures 1 and 2. The outline of the defect was delineated on the skin. Then, a midline of the defect was depicted. Subsequently, the straight lines vertical to the midline in the 1.5 cm interval were drawn to 2.0 cm outside the lateral defect edges. Small skin incisions on both ends of the vertical lines were made. In each incision, a sufficient space for subsequent ligation in the subcutaneous fat tissue was made using a mosquito Pean. Then, #1 Surgilon® needleless thread (Covidien, Dublin, Ireland) was passed through the incision on an end of the vertical line into the abdomen using a suture‐passer (EndoClose®, Covidien) (Figure 1A, Figure 2A). Subsequently, the thread in the abdomen was exteriorized through the incision on the contralateral end of the same vertical line using the suture‐passer. Special attention must be paid to the direction of the suture‐passer so as to vertically penetrate the abdominal wall (Figure 1A,B, Figure 2A). At this stage, both ends of the threads were exteriorized from both ends of the vertical lines (Figures 1B and 2B). Then, the suture‐passer was penetrated subcutaneously from one end of the vertical line toward the contralateral end (Figures 1C and 2C). Then, the thread exteriorized from the contralateral end was passed subcutaneously to the other end, from which the suture‐passer was entered toward the contralateral end. Then, both ends of the thread were exteriorized from the same incision (Figures 1D and 2D). Namely, the exit for the thread was switched from one end to the other end. Thus, we call this technique the subcutaneous switching suture technique. After switching all threads, a mesh coated with an absorbable hydrogel barrier (Ventralight ST®, C. R. BARD, Inc, Franklin Lakes, NJ, USA) was inserted into the abdomen (Figure 3A).Subsequently, the threads were ligatured tightly, achieving the defect closure (Figure 3B). Then, the mesh was spread and fixed to the abdominal wall (Figure 3C,D).

Between April 2016 and December 16, 2019 LVIHRs using this technique were performed. The patient demographics and outcomes are summarized in Table 1. Median operation time was 78 minutes (range, 49‐310 minutes). The median length and width of the defect was 10.5 cm (range, 4.0‐18.0 cm) and 7.5 cm (range, 4.0‐15.0 cm), respectively. Early and mid‐term postoperative pain was a sole Clavien‐Dindo grade II complication with 38% (6/16) incidence. Any grade III or more severe complications were not observed. Postoperative pain was evaluated by the numeric rating scale for pain intensity (NRS‐PI).^10^ Within a week after surgery, postoperative pain was severe with a median NRS‐PI of 7 (range, 3‐10). However, the pain was thereafter relieved gradually with a median NRS‐PI of 1 (range, 0‐3) up to 3 months after surgery. After that, the pain was nearly entirely eradicated with a median NRS‐PI of 0 (range, 0‐1). Postoperative follow‐up period ranged 5‐46 months with a median of 25. Fortunately, surgical site infection, seroma, mesh infection, mesh bulging, chronic pain, or recurrence was not observed in any of the patients. All patients currently declare their well‐being and express their feeling that they are satisfied with receiving the IPOM‐plus.

**TABLE 1:** Characteristics of the patients

Variables	Data
Demographics	
Age at surgery	59 years (32‐84)
Gender (male/ female)	12/ 4
Hernia characteristics	
Defect length (cm)	10.5 (4.0‐18.0)
Defect width (cm)	7.5 (4.0‐15.0)
Intraoperative variables	
Operation time (min)	78 (49‐310)
Blood loss (mL)	3 (0‐50)
Postoperative variables	
Length of stay (d)	3 (0‐7)
Early postoperative grade II complications	37.5% (6/ 16)
Pain	6 (37.5%)
Surgical site infection	0
Seroma	0
Mesh infection	0
Early postoperative grade III complications	0% (0/ 16)
Surgical site infection	0
Seroma	0
Mesh infection	0
Postoperative follow‐up period (mo)	25 (5‐46)
Long‐term complications	0% (0/ 16)
Mesh bulging	0
Chronic pain	0
Hernia recurrence	0
Chronological changes in the numeric rating scale for pain intensity	
POD 0 to 7	7 (3‐10)
POD 7 to POM 3	1 (0‐3)
POM 3 ~	0 (0‐1)

Abbreviations: POM, postoperative mo; POD, postoperative d.

## 2 DISCUSSION

Intraperitoneal‐onlay‐mesh repair without hernia defect closure (IPOM) and IPOM‐plus have become the worldwide‐prevalent techniques.^1^, ^2^, ^3^, ^4^, ^5^, ^6^, ^7^, ^8^, ^9^ Although any randomized controlled trials to compare these techniques have not been reported yet, the IPOM has been reportedly more susceptible to seroma, mesh infection, and mesh bulging than the IPOM‐plus.^3^, ^8^ The IPOM‐plus is more likely to cause severe early postoperative pain, chronic pain, and continuous abdominal discomfort than the IPOM.^1^, ^2^, ^3^, ^4^, ^5^, ^6^, ^7^, ^8^, ^9^ With emphasis on the prevention of the seroma, mesh infection, and mesh bulging, we utilized the IPOM‐plus. It has been reported that the larger the hernia defect, the greater the benefits of the defect closure.^2^, ^3^, ^4^, ^5^, ^6^, ^7^ However, the defect closure tends to be complicated in the large defect cases. Thus, we devised this technique for easy, reliable, and firm closure even in large defect cases. Although we recognize that our technique needs to be evaluated in comparison with other techniques, we could not compare this technique with others because we have only used this technique since initiating our LVIHR program. In addition, the sample size was very small. However, because any noticeable complications have not currently been observed, we consider that favorable outcomes have been obtained through our technique.

## AUTHOR CONTRIBUTIONS

Morioka, Izumisawa, Miura, and Sato had full access to all data in the study and take responsibility for the integrity of data and the accuracy of data analysis.

Study concept and design: Morioka, Izumisawa, Asano, Ohyama, and Sato.

Acquisition of Data: Izumisawa, Ohyama, Asano, Yamaguchi, Horii, Morioka, and Sato.

Analysis and interpretation of data: Asano, Yamaguchi, Horii, Morioka, Miura, and Sato.

Drafting of the manuscript: Morioka, Izumisawa, Miura, and Sato.

Critical revision of the manuscript for important intellectual content: Morioka, Izumisawa, Ohyama, Asano, Yamaguchi, Horii, Miura, and Sato.

Study supervision: Izumisawa, Ohyama, Miura, and Sato.
